# Not every twist is ovarian torsion: a case report of isolated torsion of the fallopian tube in a child

**DOI:** 10.1097/MS9.0000000000001059

**Published:** 2023-07-08

**Authors:** Shila Awal, Pradeep Raj Regmi, Nabin Prajapati

**Affiliations:** a Suryabinayak Municipal Hospital, Bhaktapur; b Department of Radiology, Tribhuvan University Teaching Hospital, Kathmandu, Nepal

**Keywords:** adnexal radiology, adnexal torsion, fallopian tube torsion, lower abdominal pain

## Abstract

Isolated fallopian tube torsion is a rare condition presenting with abdomen pain. The diagnosis is challenging because the clinical findings mimic several other conditions. In this article, we present a case of an adolescent girl who was diagnosed with the condition during laparoscopy.

**Presentation of the case:**

A 15-year-old girl presented with complaints of sudden onset left lower quadrant pain, nausea, and vomiting. There was tenderness in the left iliac fossa. Ultrasonography revealed inconclusive findings and contrast-enhanced computed tomography of the abdomen and pelvis suggested possible left adnexal torsion. Hence, she underwent a diagnostic laparoscopy which revealed a twisted, edematous, and congested left fallopian tube. The diagnosis of isolated left fallopian tube torsion was made and she was managed with unilateral salpingectomy.

**Discussion:**

Women of the reproductive age group are usually affected by this condition. The common presentations are abdominal pain, nausea, and vomiting. The physical examination may reveal abdominal and cervical motion tenderness. Per abdominal ultrasound is the first go-to modality in children. Magnetic resonance imaging, if available, is advised in children if the ultrasound is inconclusive because of the absence of radiation. However, it may require sedation. Therefore, contrast-enhanced computed tomography abdomen gives an added advantage in such scenarios as in our case. This condition is managed by surgery with salpingectomy or tube detorsion with preservation of the tube, depending on the intraoperative findings.

**Conclusion:**

Clinicians should be aware of the condition given the rarity and challenges in the diagnosis of isolated fallopian tube torsion.

## Introduction

Highlights

- An isolated fallopian tube is a rare diagnosis.
- It usually presents in women of reproductive age group.
- Presentations are nonspecific.
- Surgery is the preferred choice for management.

Isolated fallopian tube torsion is a rare condition, and diagnosis is frequently missed because of nonspecific presentations mimicking other conditions, such as ovarian torsion and appendicitis^1^. Early identification of the case is important for prompt management and prevention of future complications such as infertility. Here, we present a case of an adolescent girl who was diagnosed with isolated torsion of the fallopian tube and successfully managed with laparoscopic salpingectomy. Our article has been reported in line with the Surgical CAse REport (SCARE) criteria^2^.

## Presentation of the case

A 15-year-old girl presented to our emergency department with a complaint of lower abdominal pain for 12 h. The pain was sudden at the onset and continuous. It was located in the left iliac fossa and was severe, non-radiating, and progressive. She also complained of nausea and vomiting since the onset of the pain. Her last menstrual period was 15 days before the presentation. There was no history of recent vigorous activity.

On physical examination, her vital signs were stable except that she was tachycardic with a pulse rate of 126 beats per minute. There was tenderness on the left iliac fossa; however, no adnexal mass could be palpated. There was no guarding or rigidity per abdomen. Her complete blood count, erythrocyte sedimentation rate (ESR), and biochemistry were within normal range.

**Figure 1:**
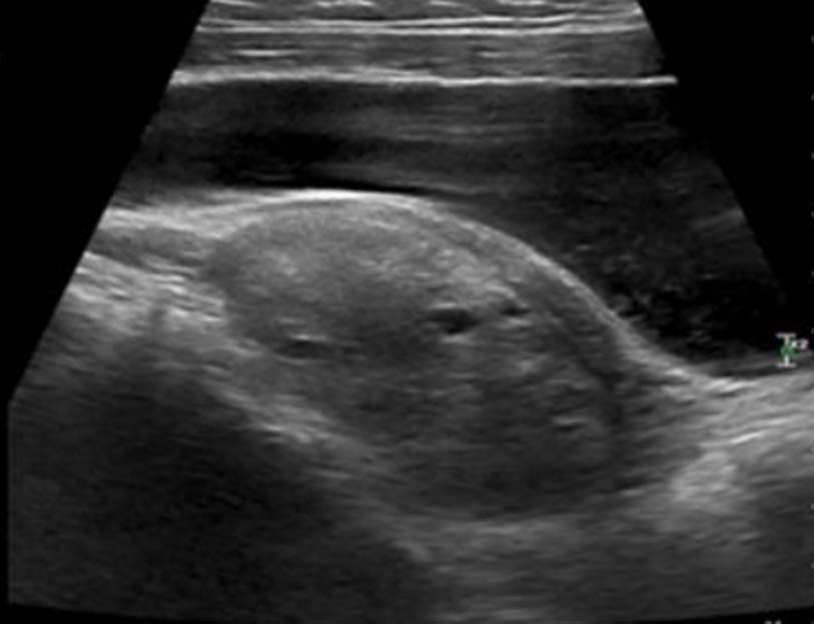
Per abdominal ultrasound image shows a heterogeneous lesion in the left adnexa with some cystic areas.

**Figure 2:**
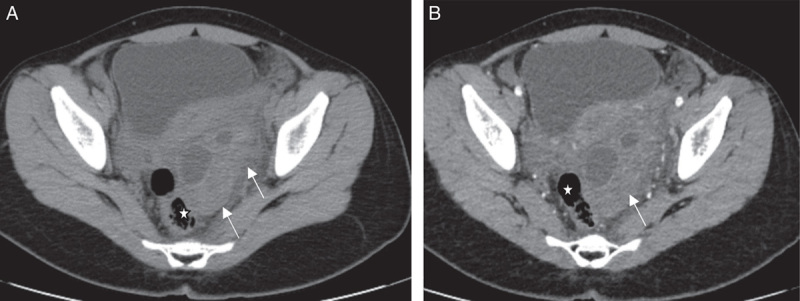
(A) Non-contrast computed tomography (CT) image shows a heterogeneous lesion with cystic spaces (white arrows) in the left adnexa, causing a mass effect to the rectum (white star). (B) Contrast-enhanced computed tomography image shows heterogeneously enhancing lesions with some cystic spaces (white arrow). This lesion can be confused with the enlarged ovary with peripherally placed follicles. Rectum is shown by a white star.

Per abdominal ultrasound (Fig. 1) shows a heterogeneous lesion in the left adnexa with some cystic areas. However, torsion could not be ruled out. A provisional diagnosis of left ovarian torsion was made. Transvaginal ultrasound was not done due to her age. In addition, the non-contrast computed tomography (CT) image (Fig. 2A) shows a heterogeneous lesion with cystic spaces (white arrows) in the left adnexa, causing a mass effect to the rectum (white star). Contrast-enhanced computed tomography image (Fig. 2B) shows heterogeneously enhancing lesions with some cystic spaces (white arrow). This lesion can be confused with the enlarged ovary with peripherally placed follicles. Rectum is shown by a white star. The diagnosis of left adnexal torsion, probably ovarian torsion, was made, and she underwent an emergency laparoscopy for the management of torsion.

**Figure 3:**
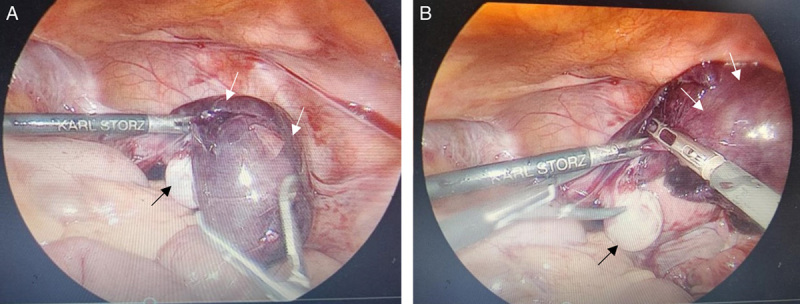
(A and B) Laparoscopic image showing enlarged reddish brown fallopian tube (white arrows) with the left ovary on the medial aspect (black arrow).

On laparoscopy, the left fallopian tube was twisted multiple times and was edematous and congested as a result of torsion (Figs 3A, B). The uterus and right adnexa were normal on examination. There were no paratubal or paraovarian cysts. A left salpingectomy was performed. The postoperative phase was uneventful; hence she was discharged after 2 days of surgery. A postoperative follow-up was scheduled after 7 days of surgery and it was uneventful.

## Discussion

Isolated fallopian tube torsion is a rare cause of lower abdominal quadrant pain. Women of reproductive age group are mostly affected^3^. Very few cases have been reported among premenarchal girls^4^. The incidence of the condition is estimated to be 1 in 1.5 million women^3^.

Factors predisposing to fallopian tube torsion can be divided into intrinsic and extrinsic. Intrinsic factors include congenital anomalies of the fallopian tube and acquired conditions such as hydrosalpinx, cysts, neoplasms, pelvic inflammatory disease, and abnormal peristalsis, whereas extrinsic factors include uterine enlargement, adnexal mass, adhesions, and trauma^1,3,4^. In our case, no predisposing factors could be identified.

The most common presenting complaint is sharp and colicky abdominal pain that can radiate to the flank or thigh^5,6^. The pain can be associated with nausea, vomiting, and bowel and bladder symptoms^6^. Physical findings usually include tender adnexal mass and cervical motion tenderness^5,7^. This is consistent with our case, as she also presented with abdominal pain, nausea, vomiting, and tenderness in the left iliac fossa. Although her laboratory findings were normal, literature has described that there may be a mild elevation in complete blood count and ESR^5^.

Imaging findings are nonspecific and hence, diagnosis is usually established only during surgery. Typical ultrasonography finding of isolated fallopian tube torsion consists of the dilated fallopian tube with thickened echogenic wall tapering toward either end, giving rise to a ‘beak sign’^8^. Doppler ultrasonography can also aid in diagnosis as impedance or absent flow in the tubular structure can be demonstrated^6,7^. CT findings include dilated and twisted tube with thickened wall and an adnexal cyst isolated from the ovary^4^. In this case, clinical and imaging findings led to a misdiagnosis of ovarian torsion, which was later found to be tubular torsion during laparoscopy.

The isolated torsion of the fallopian tube is reported to be more common on the right side because of the location of the sigmoid colon on the left side, which prevents adnexal movement^7^, dextrorotation of the uterus, and relatively increased venous flow on the left^9^. However, our case is unique since she presented with left-sided torsion.

The isolated fallopian tube torsion should be managed with prompt surgery. If the tube is not completely twisted and the presentation is of short duration, it can be salvaged. However, if the tube is necrotic, salpingectomy is the only option for the management^10^.

## Conclusion

Isolated fallopian tube torsion is a rare clinical entity. Because of the nonspecific clinical–radiologic presentation, the diagnosis is often challenging and only possible during operation. It is recommended that clinicians remain vigilant of the diagnosis for prompt management and prevention of complications.

## Ethical approval

This case report did not require review by the ethics committee.

## Patient consent

Written informed consent was obtained from the patient for the publication of this case report and accompanying images. A copy of the written informed consent is available for review by the editor-in-chief of this journal on request.

## Sources of funding

None.

## Author contribution

S.A.: contributed to the preparation of the first draft of the manuscript, review of the literature, and editing of the manuscript; P.R.R.: collected information on the case and checked and confirmed the final version of the manuscript; N.P.: contributed to the preparation of the first draft of the manuscript.

## Conflicts of interest disclosure

There are no conflicts of interest.

## Research registration unique identifying number (UIN)

Not applicable.

## Guarantor

Pradeep Raj Regmi.

## Data availability statement

Datasets generated during the current study are publicly available.

## Provenance and peer review

Not commissioned, externally peer-reviewed.

## Acknowledgements

We would like to acknowledge Professor Dr Padam Raj Panta, Dr Sandesh Poudel, Dr Atit Poudel, Dr Shree Prasad Adhikari, and Dr Ichha Upreti for providing the operative images.
